# Genome-wide characterization of *FAD* gene family in *Xanthoceras sorbifolium* Bunge and germplasm assessment

**DOI:** 10.1371/journal.pone.0318900

**Published:** 2025-03-27

**Authors:** Qinqin Xing, Huijuan He, Zhijun Wang, Yaqiang Li, Xerdiman Didar, Tao Zhou

**Affiliations:** 1 Xinjiang Academy of Forestry, Urumqi, Xinjiang, China,; 2 Research Institute of Subtropical Forestry, Chinese Academy of Forestry, Hangzhou, Zhejiang, China; South China Agricultural University, CHINA

## Abstract

Fatty acid desaturases (FADs) play a pivotal role in the accumulation of oils in plant seeds. To elucidate the role of *FADs* in oil accumulation in the seeds of *Xanthoceras sorbifolium*, this study employed bioinformatics analysis methods to identify and analyze the *FAD* gene family. A total of 25 *XsFAD* genes were unevenly distributed across 11 chromosomes. Systematic phylogenetic analysis revealed that the *XsFAD* gene family is divided into three branches, with each branch exhibiting similar gene structures. The regulatory elements within the *XsFAD* gene promoter indicate that *XsFAD* genes are regulated by multiple factors. Quantitative real-time polymerase chain reaction (qRT-PCR) validation revealed a positive correlation between the expression levels of *XsFAD* genes and the oil content of *X. sorbifolium*. By conducting phenotypic measurements of the *X. sorbifolium* fruit and seeds, as well as quantitative analysis of the *XsFAD* gene expression, it has been preliminarily identified that the Liudong-5 variety may poseess the potential to be developed into a high-yield oil-producing variety, which may be related to the habitat of the *X. sorbifolium* germplasm and requires further investagation. In summary, this study provides a foundational understanding of the *FAD* gene family of *X. sorbifolium*, and the research outcomes will contribute to the theoretical basis for the selection of high-yielding oil varieties of *X. sorbifolium* in the Xinjiang region, as well as further genetic breeding and cultivation system studies.

## 1. Introduction

### 1.1. *FAD* gene functions

Fatty Acid Desaturase (FAD) is a key enzyme in the biosynthesis of polyunsaturated fatty acids (PUFAs) in plants, catalyzing the dehydrogenation of C-C at specific positions on fatty acid chains to form C = C [1], thereby generating PUFAs with varying double bond positions and numbers. These PUFAs are essential for numerous biological processes, including the formation and stability of plant cell membranes, signal transduction, and resistance to environmental stresses. The *FAD* gene family is widely distributed and abundant in plants, typically featuring multiple exons and introns with complex and highly conserved structures.

The expression of *FAD* genes is tissue-specific and developmentally regulated, often displaying differential expression patterns in different tissues or developmental stages [2,3]. Environmental factors such as light, temperature, and water stress can significantly influence the expression levels of *FAD* genes, thereby regulating the synthesis and accumulation of PUFAs in plants. Recent studies have concentrated on the specific functions and mechanisms of *FAD* genes in plants through cloning, expression analysis, and functional validation [4]. In response to environmental stresses such as drought, salinity, and high and low temperatures, plants frequently adjust the expression of *FAD* genes. For instance, in *Cicer arietinum*, drought priming mitigates membrane damage and chlorophyll degradation by increasing membrane unsaturated fatty acids (UFAs) alongside the upregulation of various fatty acid desaturase (*CaFAD*) genes and antioxidative machinery during flowering, which improves seed yield in PBG5 [5]. In tobacco, overexpression of *CbFAD3* results in a sustained increase in linolenic acid (LA) in leaves and roots, enhancing membrane fluidity and stability and improving plant tolerance to cold, drought, and salt stress [6]. *FAD* genes may also participate in the response of plants to pathogen infection [7,8], heavy metal pollution, and other stresses. Given the significant role of *FAD* genes in plant fatty acid synthesis and stress response, they have extensive applications in crop breeding. By genetically engineering *FAD* genes for targeted modification and expression regulation, it is possible to develop crop varieties with superior fatty acid composition and resistance to adverse conditions. For instance, overexpression of *FAD* genes can increase the content and proportion of PUFAs in crop seeds, improving the nutritional value and health benefits of edible oils [9]. Conversely, knocking out or suppressing the expression of *FAD* genes can reduce the sensitivity of crops to unfavorable environmental conditions, enhancing their resistance and yield [10].

### 1.2. *Xanthoceras sorbifolium* characteristics

*Xanthoceras sorbifolium*, a member of the Sapindaceae family, is a plant species that is widely distributed across northeastern, northwestern, and northern China. With a large adaptive range, it facilitates easy reproduction and exhibits tolerance to drought and nutrient-poor. It is well-suited for cultivation in Chinese farmland, which has rich mountainous resources. The oil content of *X. sorbifolium* seeds ranges from 50% to 67%, with high levels of unsaturated fatty acids, including oleic acid (52.8% to 53.3%), linoleic acid (37.8% to 39.4%), and other fatty acids such as eicosapentaenoic acid, palmitic acid, and linolenic acid. Its oil is rich in nutrients and is an ideal raw material for producing high-quality edible oils and biodiesel. *X. sorbifolium* oil also has significant medicinal value, demonstrating preventive and therapeutic effects on cardiovascular diseases [11]. Qiang Liang et al. have assembled a high-quality genome sequence of *X. sorbifolium* using advanced technologies such as Illumina sequencing, Pacific Biosciences (PacBio) single-molecule real-time sequencing, 10X Genomics linked reads, Bionano optical maps, and high-throughput chromosome conformation capture (Hi-C) [2]. The assembled genome size is 439.97 Mb, with 15 pseudo-chromosomes accounting for 95.42% (419.84 Mb) of the assembled genome, and 56.39% of the genome is repetitive. This genome contains 21,059 protein-coding genes, from which multiple genes with tissue-specific expression patterns have been identified. However, the *FAD* gene family in these oil plants has not been well studied, and little is known about the molecular systematics and differential expression characteristics of the *FAD* gene family across different growth regions. By studying the *FAD* gene family of *X. sorbifolium*, the *FAD* gene related to seed oil content can be identified, providing a basis for subsequent genetic breeding to improve the oil content of *X. sorbifolium*.

### 1.3. The study objectives

Therefore, this study aims to analyze the structure, physical properties, and expression patterns of *FAD* genes in *X. sorbifolium*, to further understand the molecular evolutionary characteristics of the *XsFAD* gene family. Moreover, as the main distribution area of *X. sorbifolium* in the western region of China, Xinjiang holds a wealth of local, wild, and introduced superior germplasm resources. The significant differences in environmental and climatic conditions have led to the accumulation of substantial genetic variation during the evolutionary process, obtaining the best varieties through the analysis of germplasm resources. This lays the foundation for breeding varieties with high economic value and specific traits and serves as a reference for genetic improvement research focusing on oil content, stress tolerance, and disease resistance in *X. sorbifolium*.

## 2. Materials and methods

### 2.1. Identification of *XsFAD* genes

To identify the *FAD* genes in *X. sorbifolium*, we downloaded the genome data, protein files, and gff3 annotation files (GCA_020796215.1) from the NCBI database (https://www.ncbi.nlm.nih.gov/). Utilizing the Hmmer-search 3.0 program to search protein sequences containing FAD domains (PF00487, PF03405, and PF10520) within the *X. sorbifolium* protein database, with a search threshold of E < 1e^-10^. Submitting the preliminarily selected *X. sorbifolium* FAD protein sequences to the SMART (http://smart.embl-heidelberg.de/), NCBI-CDD (https://www.ncbi.nlm.nih.gov/cdd/), and PFAM (http://pfam.xfam.org/) websites to ensure they possess complete FAD domains. After removing suspicious sequences, obtain the members of the *XsFAD* gene family. The *XsFAD* gene family members were named according to their physical location order on the 15 chromosomes of *X. sorbifolium*. Molecular weight (MW) and isoelectric point (PI) were predicted in Expasy (https://web.expasy.org). Using BUSCA (http://busca.biocomp.unibo.it/) to predict the subcellular localization of the *XsFAD* genes encoded proteins. Predicting the localization of XsFAD-encoded proteins helps us understand the specific biological processes they are involved in and can identify other proteins that they interact with.

### 2.2. Phylogenetic tree construction of *XsFAD* genes

The protein sequences of the *XsFAD* gene family members were aligned using ClustalW v2.1. The phylogenetic tree was constructed using MEGA 11 with the Neighbor-Joining method. The phylogenetic tree was edited and beautified using the iTOL 2.0 website (https://itol.embl.de/).

### 2.3. Analysis of cis-acting elements of *XsFAD* genes

Using TBtools, sequences of the 2000 bp region upstream of the ATG start codon of the *XsFAD* gene family genes were extracted based on the genomic annotation and sequence files of *X. sorbifolium*. These sequences were submitted to the PlantCARE website (http://bioinformatics.psb.ugent.be/webtools/plantcare/html/) for analysis of promoter cis-acting elements. The analysis results were organized and imported into TBtools for visualization.

### 2.4. Chromosomal location analysis of *XsFAD* genes

With TBtools, the lengths of each chromosome, gene density, and chromosomal localization information of the *XsFAD* genes were extracted from the plant genome database and didn’t include genetic distance for the creation of a chromosomal localization map of the *XsFAD* genes.

### 2.5. Analysis of conserved motifs and gene structure of *XsFAD* genes

The MEME online tool (http://meme-suite.org/) was used to analyze the conserved motifs in the XsFAD proteins, with the number of motifs set to 10. The GSDS online tool (http://gsds.cbi.pku.edu.cn/) was used to analyze the gene structures of *XsFAD* genes, and the obtained files were imported into TBtools for visualization.

### 2.6. Gene duplication relationships and synteny analysis of *XsFAD* genes

To study duplication events of *XsFAD* genes, MCScanX was used for collinearity analysis. The extracted gene collinearity results were visualized using TBtools to generate an *XsFAD* gene family collinearity map.

### 2.7. Selection pressure analysis of *XsFAD* genes

ClustalX (http://www.clustal.org/) was used to align the *XsFAD* gene sequences, and KaKs_Calculator 3.0 (https://ngdc.cncb.ac.cn/biocode/tools/BT000001/releases/3.0) was utilized to analyze the selection pressure between gene pairs.

### 2.8. Protein interaction network analysis of *XsFAD* genes

STRING (https://string-db.org/) was used to predict the protein interactions of the XsFAD-encoded proteins, establishing the corresponding protein relationship network and visualizing it.

### 2.9. Investigation of plant germplasm and oil content analysis

In this study, 10 germplasm resources of the plant were obtained from the Xinjiang Academy of Forestry Sciences. These germplasm were located in various regions of Xinjiang, China: Korla (41° 43′ 48″ N, 86° 1′ 12″ E, Germplasm ID: Liudong-1), Qitai County (44° 1′ 12″ N, 89° 30′ 36″ E, Germplasm ID: 131-75), Urumqi (43° 49′ 11.99″ N, 87° 36′ 36″ E, Germplasm ID: Liudong-5), Karamay (45° 36′ 0″ N, 84° 53′ 24″ E, Germplasm ID: 80 acres-1), Changji (44° 48′ 0″ N, 87° 12′ 0″ E, Germplasm ID: 80 acres-5), Kizilsu (44° 4′ 48″ N, 87° 16′ 12″ E, Germplasm ID: 80 acres-7), Aksu (41° 12′ 0″ N, 80° 12′ 0″ E, Germplasm ID: 49-4), Manas County (44° 12′ 0″ N, 86° 6′ 36″ E, Germplasm ID: 80-3), Ili (43° 54′ 36″ N, 81° 16′ 48″ E, Germplasm ID: 80-3-2), and Kuqa (41° 45′ 0″ N, 82° 56′ 24″ E, Germplasm ID: 81-6-1). The investigation included the shape, cracking mode, average length, and average diameter of the fruits under the same growth period. Furthermore, the oil content of the seeds was measured using the method referenced by Gu Yao et al. [12]. Seed oil is obtained by drying, grinding, leaching, filtering, and concentrating, and it is weighed to calculate the oil yield. Each variety was analyzed in triplicate.

### 2.10. Comprehensive assessment of germplasm from different regions

The original data of the 11 growth characteristics were listed in the original data table (Table S1). The maximum value for each indicator was identified as the standard value, normalizing it to a value of 1. Each value in that indicator column was divided by the standard value, and the resulting quotients were filled into the “Matrix Coordinate Table” (Table S2). The squared deviation sum of the values in each column of the matrix coordinate table relative to other standard values was calculated as $p_{i}^{2}={\left(1-a_{ij}\right)}^{2}$ , which was filled into the *P*^2^*i* comprehensive assessment table. The row sums provided the squared sums of the inter-point distances for each indicator: $\sum p_{i}^{2}=\sum {\left(1-a_{ij}\right)}^{2}$. The evaluation order was determined according to the size of $\sum p_{i}^{2}$ [13].

### 2.11. Expression analysis of *XsFAD* genes in germplasm from different regions

Total RNA from the seeds of each variety was extracted using the RNAprep Pure Plant Total RNA Extraction Kit (DP432). The expression levels of relevant genes were detected using quantitative real-time polymerase chain reaction (qRT-PCR). The ABI 7500 fluorescence quantitative PCR detection system (Thermo Fisher Scientific Inc., Massachusetts, USA) was utilized according to the operational steps of Takara’s One Step TB Green® PrimeScript™ RT-PCR Kit II (Takara Bio Inc., Tokyo, Japan), with glyceraldehyde-3-phosphate dehydrogenase (GAPDH) as the internal reference gene. Each sample was biologically repeated three times, and the relative expression levels of each gene were calculated using the $2^{-��C_{t}}$. The primers were designed using primer 5.0, the primer information is shown in Table 1.

**Table 1:** QRT-PCR Gene annotation and primer design.

**Gene ID**	**Forward primer (5**′** → 3**′**)**	**Reverse primer (5**′** → 3**′**)**
**Xs*FAD*9**	CAATGGCCTCCACTCTTCGT	CTGAGGCTCATTTGCGGAGA
**Xs*FAD*18**	GGGTTGGCACAACAACCATC	GCCAAACCAATTGCCTGGAG
**Xs*FAD*6**	CAATGGCCTCCACTCTTCGT	CTGAGGCTCATTTGCGGAGA

### 2.11. Statistical analysis

ANOVA of gene expression and oil content data was conducted by SPSS Statistics 23, with p < 0.05 as the basis for statistical differences, and the graph was drawn through origin 2018.

## 3. Results

### 3.1. Identification and physicochemical property analysis of *FAD* genes in *X. sorbifolium*

Through the analysis of the whole-genome data of *X. sorbifolium*, 26 candidate genes containing *FAD* domains were identified, among which 25 members matched the characteristics of FAD family proteins. These 25 members were validated through the SMART, NCBI-CDD, and PFAM websites. The sequence was named *XsFAD1*-*XsFAD25* based on their physical location on the chromosomes. The physicochemical property analysis of XsFAD-encoded proteins showed a sequence length range of 346-8,291 amino acids, a molecular weight distribution of 7,834.91-56,868.51 Da, and an isoelectric point range of 5.34-9.55. The predicted subcellular localization of XsFAD-encoded proteins includes chloroplasts, cytoplasm, and the endoplasmic reticulum (Table 2). These findings laid a foundation for further functional research and exploration of the role of these genes in biological processes.

**Table 2:** Physiological and biochemical characteristics of XsFAD proteins.

Gene name	Gene ID		Chromosome	Start site	End site	Length/AA	MW(Da)	pl	Subcellular localization
**Xs*FAD*1**	JRO89_XS01G0325300		chr1	31942976	31944319	1,344	51,672.47	8.75	Plasma membrane
**Xs*FAD*2**	JRO89_XS03G0033600		chr3	3820998	3827174	6,177	45,512.94	6.24	Chloroplast
**Xs*FAD*3**	JRO89_XS03G0321200		chr3	33889151	33895720	6,570	38,601.64	5.34	Cytoplasm
**Xs*FAD*4**	JRO89_XS03G0322000		chr3	33979898	33982457	2,560	49,844.95	5.58	Chloroplast
**Xs*FAD*5**	JRO89_XS03G0322100		chr3	33993831	33995031	1,201	40,527.15	5.77	Cytoplasm
**Xs*FAD*6**	JRO89_XS03G0322200		chr3	34000463	34004125	3,663	46,068.46	6.25	Chloroplast
**Xs*FAD*7**	JRO89_XS04G0192000		chr4	25290634	25291338	705	26,086.76	6.17	Cytoplasmic
**Xs*FAD*8**	JRO89_XS04G0258200		chr4	31153201	31154547	1,347	51,849.02	9.07	Plasma membrane
**Xs*FAD*9**	JRO89_XS04G0258300		chr4	31174367	31175857	1,491	56,868.51	9.14	Plasma membrane
**Xs*FAD*10**	JRO89_XS06G0076500		chr6	6706659	6708932	2,274	38,247.01	7.91	Plasma membrane
**Xs*FAD*11**	JRO89_XS06G0121800		chr6	11249291	11255541	6,251	51,315.84	9.31	Chloroplast
**Xs*FAD*12**	JRO89_XS06G0207700		chr6	23033587	23036630	3,044	43,843.63	5.99	Chloroplast
**Xs*FAD*13**	JRO89_XS06G0207800		chr6	23057494	23064870	7,377	11,194.77	5.86	Chloroplast
**Xs*FAD*14**	JRO89_XS06G0207900		chr6	23064928	23065273	346	7,834.91	6.82	Cytoplasmic
**Xs*FAD*15**	JRO89_XS06G0209900		chr6	23338801	23347091	8,291	48,819.28	6.52	Chloroplast
**Xs*FAD*16**	JRO89_XS07G0210900		chr7	21017050	21018418	1,369	44,050.63	6.61	Chloroplast
**Xs*FAD*17**	JRO89_XS07G0273200		chr7	28098277	28098848	572	14,324.25	5.74	Cytoplasmic
**Xs*FAD*18**	JRO89_XS09G0113800		chr9	16117001	16122137	5,137	47,359.89	9.55	Plasma membrane
**Xs*FAD*19**	JRO89_XS10G0024800		chr10	2559732	2560883	1,152	43,822.25	8.33	Endoplasmic reticulum
**Xs*FAD*20**	JRO89_XS12G0067300		chr12	5386229	5388879	2,651	50,843.98	8.51	Chloroplast
**Xs*FAD*21**	JRO89_XS12G0153500		chr12	13839899	13841050	1,152	44,484.28	8.79	Endoplasmic reticulum
**Xs*FAD*22**	JRO89_XS13G0098200		chr13	8876631	8880074	3,444	44,562.04	7.1	Endoplasmic reticulum
**Xs*FAD*23**	JRO89_XS14G0111800		chr14	10357947	10361269	3,323	51,860.2	8.99	Chloroplast
**Xs*FAD*24**	JRO89_XS15G0111900		chr15	12717199	12717897	699	25,826.53	6.52	Chloroplast
**Xs*FAD*25**	JRO89_XS15G0157000		chr15	16579245	16580186	942	34,869.75	7.78	Chloroplast

### 3.2. Phylogenetic tree of *XsFAD* gene family

**Fig. 1:**
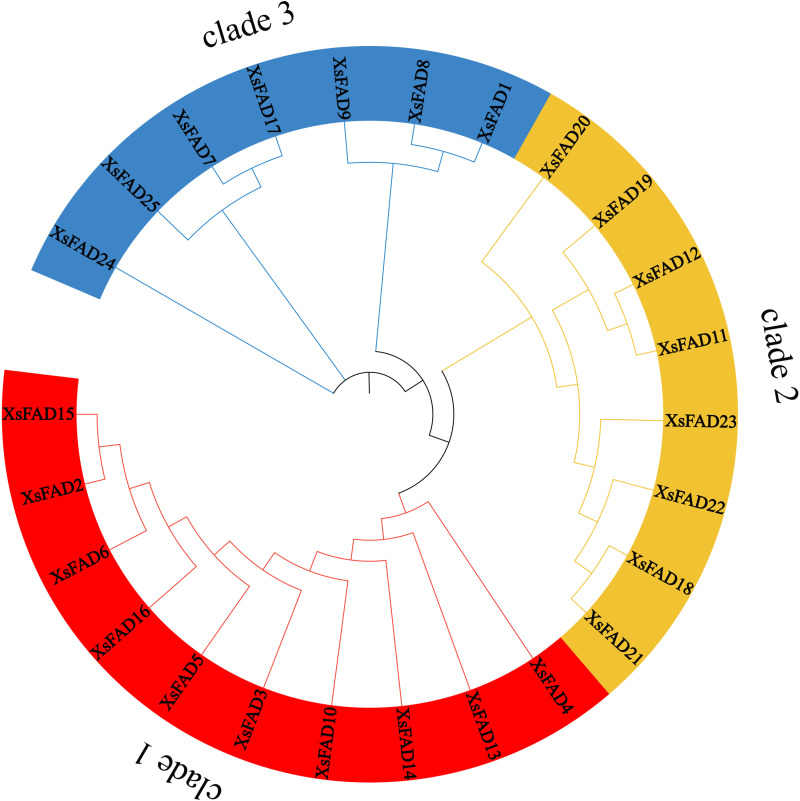
Phylogenetic tree of *XsFAD* Gene family.

The 25 XsFAD protein sequences of *X. sorbifolium* were used to construct a phylogenetic tree based on their homology (Fig. 1). The results showed that the *FAD* genes of *X. sorbifolium* can be divided into three branches, with clade 1 containing 10 *FAD* genes at the base of the phylogenetic tree, clade 2 containing 8 *FAD* genes, and clade 3 containing 7 *FAD* genes. This clustering suggests potential functional similarities and evolutionary relationships among the *FAD* genes within the species.

### 3.3. Promoter cis-acting element analysis of *XsFAD* genes

**Fig. 2:**
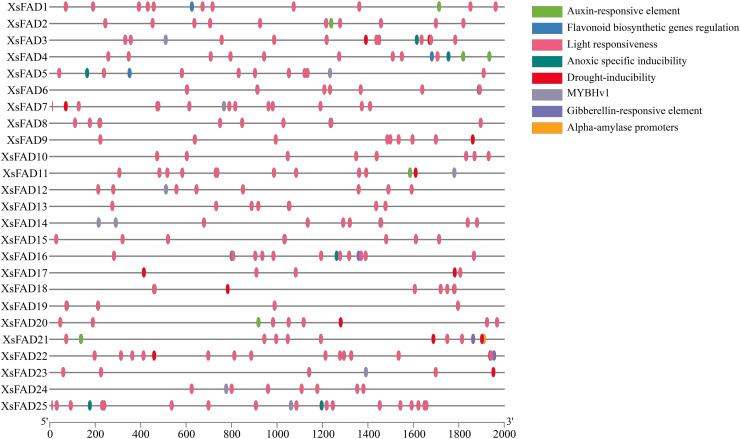
Cis-acting elements of *XsFAD* Genes promoter.

To understand the role of *XsFAD* genes in stress response and signal transduction in *X. sorbifolium*, the cis-acting elements in the 2000 bp upstream of the ATG start codon in the promoter region of the *25 XsFAD* genes were analyzed using the PlantCARE website. The results showed that the promoter region of *XsFAD* genes contains a series of important growth and abiotic stress response elements, such as ARE, MYB, and drought-inducibility (Fig. 2). Notably, the promoter regions of *XsFAD1*, *XsFAD2*, *XsFAD4*, *XsFAD11*, *XsFAD20*, and *XsFAD21* contain response elements to antioxidant reactions. Studies suggested that regulatory elements in *FAD* promoters, such as WRI1, can influence oil content by modulating gene expression through interactions with enhancers and environmental conditions like darkness, ethylene, and temperature [14], indicating that *XsFAD* promoter may participate in environmental stress responses.

### 3.4. Chromosome localization analysis of *XsFAD* genes

**Fig. 3:**
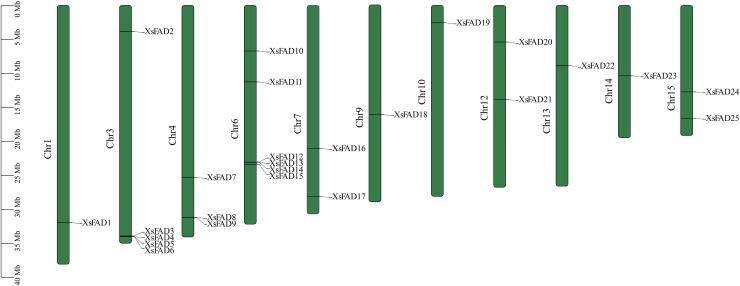
Chromosome location of *XsFAD* genes.

As shown in Fig. 3, the 25 *XsFAD* genes are unevenly distributed on 11 chromosomes of *X. sorbifolium*, with the densest distribution on chromosomes 3 and 6, each containing 5 and 6 genes, respectively. The number of *XsFAD* genes on chromosomes 1, 4, 7, 9, 10, 12, 13, 14, and 15 is 1, 3, 2, 1, 1, 2, 1, 1, 2, respectively, while no *XsFAD* genes were found on chromosomes 2, 5, 8, and 11. The uneven distribution of *XsFAD* genes across chromosomes suggests potential genomic hotspots for *FAD* gene evolution and regulation in *X. sorbifolium*.

### 3.5. Analysis of conserved motifs, domain and gene structure of *XsFAD* genes

**Fig. 4:**
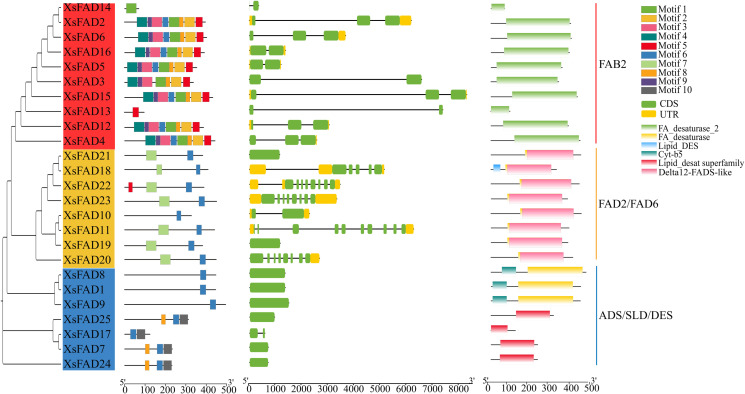
Analysis of *XsFAD* conserved Motif, genes structure, and conserved domain.

Based on evolutionary analysis, the sequence characteristics of the 25 *XsFAD* genes in *X. sorbifolium* were analyzed for their conserved motifs and gene structure features. The analysis of conserved motifs showed that the proteins in the same branch of *XsFAD* genes have similar motif compositions (Fig. 4), with motif 5 in all 9 proteins of clade 1, and motif 1, motif 2, motif 3, motif 4, motif 5, motif 8, and motif 9 in all 9 proteins, motif 6 and motif 7 in all 8 proteins of clade 2, motif 6 being present in all 7 proteins of clade 3. It can be seen that clade 1 members have more motif types and more regular motif compositions. The analysis of gene structure showed that *XsFAD* genes can contain up to 2 exons, with 8 genes lacking exons. Among these, proteins in clade 3 do not have exons. The absence of exons may be attributed to a combination of genetic mutations, evolutionary processes, alternative splicing, and gene regulation mechanisms.

By predicting the conserved domain of *FAD* genes, we found that *FAD* genes contain several conserved domains. Ye et al. studied the *FAD* gene family of *Camellia oleifera* and found that membrane-bound FADs can be further classified into five subfamilies, including FAB2, FAD4, FAD2/FAD6 (Omega-6 desaturases, ω6), FAD3/FAD7/FAD8 (Omega-3 desaturases, ω3), and ADS/SLD/DES, and identified conserved domains, including FA_desaturase, Lipid_DES, cyt-b5l, and TMEM189_B [15]. By analyzing the conserved domains of the *XsFAD* gene, clade 1 can be identified as FAB2, which contained one lengthy FA_desaturase_2 domain; clade 2 can be identified as FAD2/FAD6, including Delta12-FADS domain; the clade 3 can be identified as ADS/SLD/DES, which includes the FA_desaturase domain. This comprehensive analysis provides insights into the evolutionary relationships, motif conservation, gene structure variation, and conserved domain composition of *XsFAD* genes in *X. sorbifolium*.

### 3.6. Analysis of gene duplication and colinearity of *XsFAD* genes

**Fig. 5:**
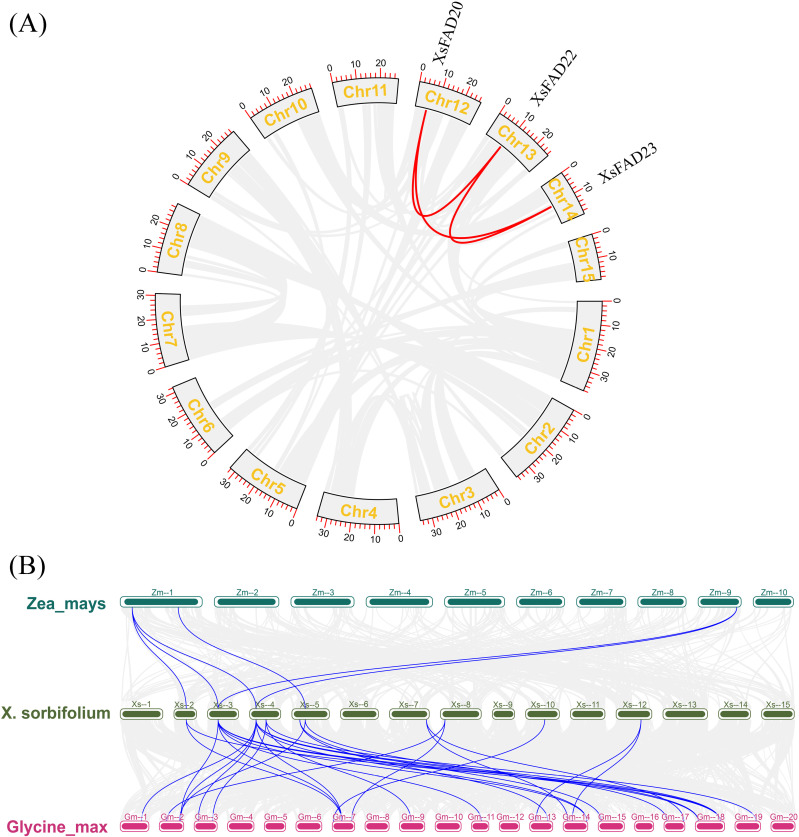
Collinearity analysis of *XsFAD* genes in *X. sorbifolium.* (A) Collinearity analysis of genes within species. (B) Collinearity analysis of genes between species.

To further analyze the evolutionary relationship of *FAD* genes in *X. sorbifolium*, the gene duplication events of the 25 *XsFAD* genes were analyzed. The results showed that there are three pairs of colinear genes among *XsFAD* genes: *XsFAD20*/*XsFAD22*, *XsFAD20*/*XsFAD23*, and *XsFAD22*/*XsFAD23*, indicating that the *XsFAD* gene family has undergone gene duplication events (Fig. 5A). By analyzing the gene collinearity of *X. sorbifolium*, *Zea* mays and *Glycine* max, we found that there are three genes collinear with *XsFAD* genes in maize and 21 genes collinear with *XsFAD* genes in soybean, which may have similar functions to *XsFAD* genes (Fig. 5B). The identification of colinear gene pairs highlights potential mechanisms contributing to the expansion and diversification of the *FAD* gene family within the *X. sorbifolium* genome.

### 3.7. Analysis of selection pressure on *XsFAD* genes

To study the pressure on *XsFAD* genes during evolution, a selection pressure analysis was conducted, revealing that there are eight pairs of genes under selection pressure, with their Ka/Ks values all being less than 1. This indicates that these gene sequences have fewer non-synonymous mutations than synonymous mutations, thus experiencing purifying selection. Purifying selection is important for maintaining the functionality of genes, as it helps to eliminate deleterious mutations that could disrupt the protein’s structure or function (Table 3)[16]. The prevalence of purifying selection in *XsFAD* genes underscores the evolutionary conservation and functional significance of these genes in *X. sorbifolium*.

**Table 3:** Analysis of selection pressure between *XsFAD* genes.

Sequence	Ka	Ks	Ka/Ks	Effective Lenth	Average S-sites	Average N-sites	cN	cS	pN	pS	Divergence-Time (MY)
**Xs*FAD*3&Xs*FAD*5**	0.038	0.093	0.407	1,011	227.917	783.083	29.000	20.000	0.037	0.088	3.111
**Xs*FAD*3&Xs*FAD*12**	0.023	0.144	0.163	1,011	229.333	781.667	18.000	30.000	0.023	0.131	4.792
**Xs*FAD*3&Xs*FAD*15**	0.022	0.155	0.143	1,011	229.167	781.833	17.000	32.000	0.022	0.140	5.150
**Xs*FAD*4&Xs*FAD*5**	0.036	0.117	0.306	1,065	239.167	825.833	29.000	26.000	0.035	0.109	3.915
**Xs*FAD*4&Xs*FAD*12**	0.049	0.166	0.294	1,137	258.417	878.583	41.500	38.500	0.047	0.149	5.536
**Xs*FAD*4&Xs*FAD*15**	0.092	0.226	0.406	1,230	283.667	946.333	81.667	55.333	0.086	0.195	7.531
**Xs*FAD*12&Xs*FAD*15**	0.001	0.008	0.147	1,161	264.667	896.333	1.000	2.000	0.001	0.008	0.253
**Xs*FAD*24&Xs*FAD*25**	0.042	0.078	0.547	696	162.833	533.167	22.000	12.000	0.041	0.074	2.586

### 3.8. Protein interaction network analysis of *XsFAD* genes

**Fig. 6:**
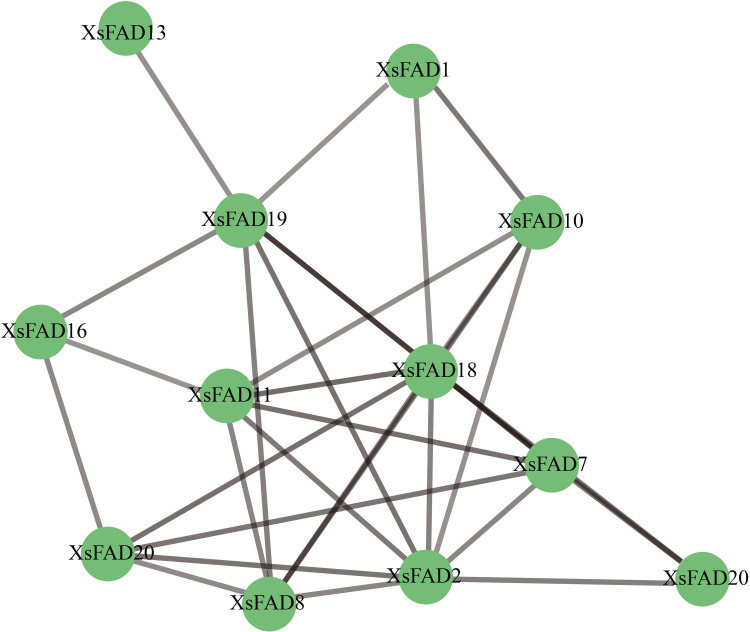
Protein interaction network analysis of *XsFAD* genes.

To analyze the possible protein interactions of XsFAD-encoded proteins in *X. sorbifolium*, the homologous genes were used to construct a protein interaction network using the STRING software. The results showed that there may be various regulatory relationships among XsFAD-encoded proteins, with XsFAD2, XsFAD7, XsFAD8, XsFAD11, XsFAD18, XsFAD20 interacting with multiple XsFAD-encoded proteins, indicating potential functional collaboration among XsFAD proteins and their coordinating role in biological pathways and processes (Fig. 6).

### 3.9. Germplasm survey and oil content analysis of *X. sorbifolium*

**Fig. 7:**
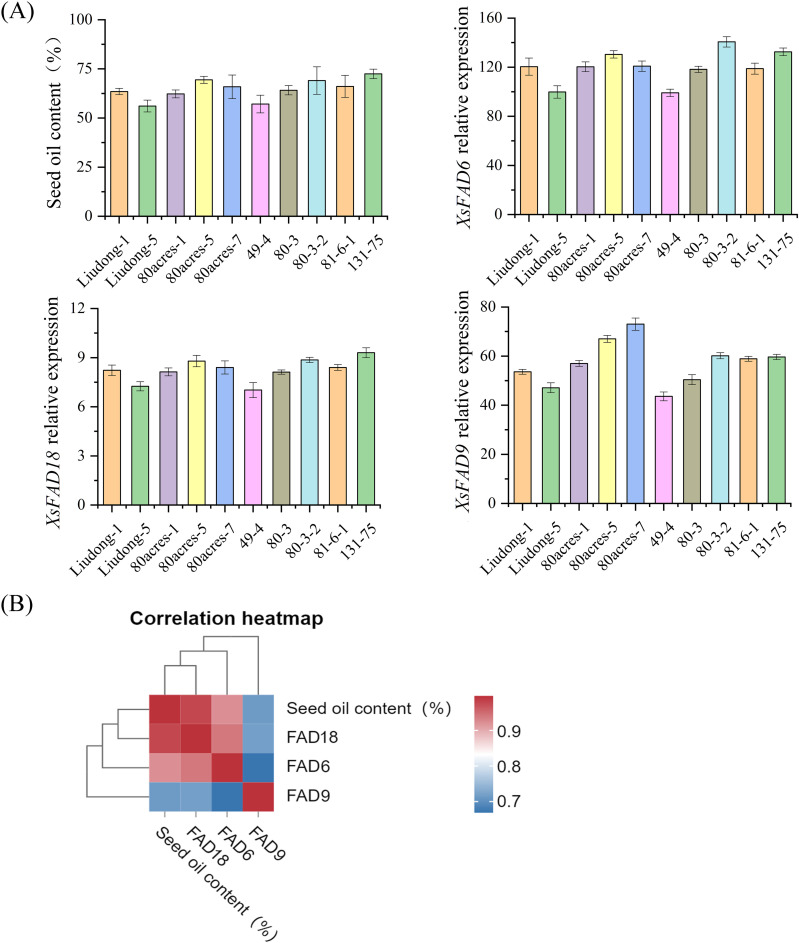
Oil content of 10 *X. sorbifolium* germplasm seeds and *FAD* gene expression. (A)Oil Content of 10 *X. sorbifolium* Germplasm Seeds and *FAD* gene expression. (B) Correlation heatmap between oil content between *FAD* gene expression.

After collecting sample information, it was found that the 10 *X. sorbifolium* germplasm fruit shapes are mainly spherical and peach-shaped, with Liudong-5 and 80-3 being cylindrical; the fruit opening method is generally 3-lobed; the largest fruit length and width are 73.03 mm and 74.17 mm for Liudong-1, respectively, and the smallest are 44.11 mm and 44.11 mm for 80 acres-1; the largest single fruit weight is 51.90 g for Liudong-1, and the smallest is 22.39 g for 80 acres-7; the largest seed diameter is 25.20 mm for 80 acres-7, and the smallest is 12.78 mm for 131–75; the highest thousand-seed weight is 1230.8 seeds for Liudong-5, and the lowest is 716 seeds for 81-6-1; the highest number of fruits is 303 for 131–75, and the lowest is 58 for 80 acres-7. In addition, the oil content of the seeds of 131-75 is the highest at 72.45%, and the lowest is 56.10% for 49-4 (Fig. 7A, Table S1). This data provides a valuable foundation for selecting high-oil content germplasm and breeding improved *X. sorbifolium* varieties tailored to specific traits and productivity goals.

### 3.10. Comprehensive evaluation of different varieties of *X. sorbifolium*

To objectively evaluate the different varieties of *X. sorbifolium* germplasm, the diameter, number, weight, and oil content of the seeds were comprehensively evaluated using the multidimensional space (Euclidean) $E^{n}$ Multi-vector theoretical comprehensive evaluation mathematical model, “coordinate comprehensive evaluation method”. According to the $\sum p_{i}^{2}$, the *X. sorbifolium* germplasm was ranked, with a higher $\sum p_{i}^{2}$ indicating poorer germplasm quality and a lower $\sum p_{i}^{2}$ indicating superior *X. sorbifolium* germplasm quality (Table S2, Table S3 ). Among the ten varieties, Liudong-5 had the highest evaluation, followed by Liudong-1 and 131-75. Liudong-5 has large fruit and seeds, with a high thousand-seed weight; although the oil content is low, the number of fruits and single plant seed weight values are high. Liudong-1 has a high number of single fruit seeds, and 131-75 has a high number of fruits, single plant seed weight, and oil content, indicating that these varieties have advantages. These three varieties of *X. sorbifolium* show promise for breeding superior cultivars through hybridization and biotechnological methods.

### 3.11. Correlation analysis of FAD gene family and oil content in *X. sorbifolium* seeds

To study the correlation between the *FAD* gene family and the oil content in *X. sorbifolium* seeds, the expression levels of three genes from each clade were detected in the seeds of different varieties of *X. sorbifolium*. The expression levels of the three *FAD* genes were significantly correlated with the oil content of *X. sorbifolium* seeds. In Liudong-5, with lower oil content, the gene expression level is also relatively low, while in 131-75, with higher oil content, the *FAD* gene expression level is relatively high. According to the correlation heatmap, we can find that seed oil content is highly correlated with the *FAD18* gene, indicating that the FAD2 clade might play an important role in oil content accumulation (Fig. 7B). These findings underscore the potential regulatory role of the *FAD* gene family in determining oil content variations among different *X. sorbifolium* varieties, offering insights into the genetic mechanisms underlying oil accumulation in *X. sorbifolium* seeds.

## 4. Discussion

Fatty acid desaturases (FADs) play a critical role in plant lipid metabolism by introducing double bonds into hydrocarbon chains, thus influencing the composition of fatty acids in oils. FAD enzymes are essential in plant growth, development, and defense mechanisms, enabling resistance against various abiotic stresses, including temperature extremes. With the advancement of genomic sequencing and molecular biology, the *FAD* gene family has been elucidated in multiple plant species, including soybean, tomato, banana, and others [1,3,7,17]. The oil of *X. sorbifolium* holds significant medicinal value, and exploring the *XsFAD* gene family can facilitate the utilization and development of this oil.

### 4.1. *XsFAD* genes phylogenetics and evolution

*XsFAD* genes encode proteins that are classified into three branches in phylogenetic analysis, suggesting that proteins within the same branch may share similar biological functions. The gene structure, conserved motifs, and domains provide evidence for the phylogenetic tree, with a cluster of genes sharing structural similarity. The sizes of *XsFAD* genes vary widely, with differences in the number of exons, indicating evolutionary divergence within the *XsFAD* gene family. Plant FADs can be divided into soluble desaturases and membrane-bound desaturases according to their solubility. Membrane-bound FADs can be further classified into five subfamilies according to their distinct functions. Fatty acid desaturase-2 (FAD2) encodes a ω-6 desaturase that functions in the endoplasmic reticulum, whereas fatty acid desaturase-6 (FAD6) encodes a ω-6 desaturase that functions in the plastids [18]. In *Arabidopsis thaliana*, three gene products of FAD3, FAD7, and FAD8 are responsible for the production of trienoic fatty acids via unsaturation at the ω-3 position [19,20]. Moreover, the FAD gene family exhibits tissue-specific expression, with *CtFAD2-1* and *CtFAD2-10* in *Carthamus tinctorius* being specifically expressed in developing seeds and flower heads, respectively, while *CtFAD2-2* shows relatively low oleic acid desaturase activity across the plant. *CtFAD2-5* and *CtFAD2-8* are specifically expressed in the root tissue of red flower seedlings, whereas *CtFAD2-3* is mainly expressed in cotyledons and hypocotyls [21]. Tomato *SlFAD* is involved in the expression of root, stem, and leaf tissues; *SlFAD8* is predominantly expressed in leaves [3]. *XsFAD* genes may also exhibit tissue specificity, with distinct functions in different tissues.

Gene duplication is a crucial mechanism for the expansion and evolution of plant gene families. Fragment and tandem duplication are considered the primary modes of gene family expansion. In this study, only three pairs of tandemly repeated genes were identified in the 14 chromosome arrays, indicating that fragment duplication played a significant role in the amplification of the *XsFAD* gene family. Similar amplification patterns have been observed in other plant gene families, such as the expansion of sweet potato (*Ipomoea batatas) CYP450* genes through tandem and fragment duplication, and the large-scale fragment repetition in the tea tree (*Camellia sinensis*) ABC gene family, with tandem duplication serving a supplementary role [22,23]. By analyzing the gene collinearity of *X. sorbifolium*, *Zea* mays and *Glycine* max, we found interesting gene conservation patterns and potential functional similarities. The identification of collinear genes in these species means that some genetic factors in FAD-related pathways are conserved. The common collinearity pattern implies the potential functional conservation or convergence of these genes in fatty acid metabolism, oil biosynthesis, or other biological processes related to FAD genes.

### 4.2. Functional insights

Transcription factors bind to cis-acting elements in the promoter regions of *XsFAD* genes, regulating gene expression. Identified transcription factors in the promoter regions of *XsFAD* genes are associated with drought resistance, antioxidant activity, light response, gibberellin response, and flavonoid synthesis, indicating that *FAD* genes can enhance the resistance of the plant to both biotic and abiotic stresses. *FADs* maintain cell membrane fluidity and integrity by altering the synthesis and ratio of unsaturated fatty acids, thus influencing plant stress tolerance [24]. For example, rice mutants lacking the ω-3 fatty acid desaturase (FADs) are unable to adapt to cold temperatures [25]. Light also affects fatty acid desaturation in plants, with dark treatment reducing the expression levels of *FAD3* and *FAD8* in soybean cell cultures and *FAD2* in olive fruits, indicating light-dependent transcriptional regulation of *FAD* genes [22,26,27]. Additionally, tomato *SlFAB5* can regulate various stages of fruit development under the influence of exogenous hormones [3]. The stress response of *XsFAD* genes to the environment will be further verified by experiments.

Fatty acid desaturase 2 (FAD2) enzymes facilitate the conversion of oleic acid (OA) to linoleic acid (LA) in plants. This crucial polyunsaturated fatty acid is a significant component of plant oils; the catalytic activity of FAD2 is closely related to the oil content in plant seeds [28]. *Artemisia sphaerocephala*, a desert shrub, is rich in linoleic acid and it possesses a large *FAD2* gene family. The proteins encoded by the *FAD2* gene family exhibit high sequence similarity and are relatively conserved during evolution. The heterologous expression in *Saccharomyces cerevisiae* demonstrates that *AsFAD2-1*, *AsFAD2-10*, and *AsFAD2-3* can convert oleic acid to linoleic acid. The *AsFAD2-1* gene is strongly expressed in developing seeds, which may be closely related to the high accumulation capacity of LA in seeds [29]. Mutations in the *FAD2-1* gene in soybeans increase the content of oleic acid in seeds [17]. By detecting the oil content in different varieties of *X. sorbifolium* and quantifying the *FAD* genes, it can be shown that the expression level of *FAD* genes is significantly correlated with the oil content of *X. sorbifolium* seeds. Among them, 131-75 has the highest oil content, and the relative expression levels of *XsFAD18* in this variety are relatively high. This indicates that *FAD2* genes can promote an increase in seed oil content.

### 4.3. Environmental adaptation

The results showed that the comprehensive evaluation of the characteristics of different varieties of *X. sorbifolium* could lay a foundation for selecting high-oil-content germplasm and cultivating improved varieties. Among them, Liudong-5 was rated as the best variety, followed by Liudong-1 and 131-75. Liudong-5 has large fruits and seeds with a high 1000-grain weight. Liudong-1 has a high number of seeds per fruit, while 131-75 is excellent in fruit number, seed weight per plant, and oil content. These varieties show the potential to cultivate excellent varieties through hybridization and biotechnology. 131-75 has a significantly higher oil content than other varieties, this may be related to environmental differences in the Xinjiang region of China, as this variety was collected from a poor area in the southern Xinjiang region, where the average annual precipitation is extremely low, and the soil pH is around 8.8-9. Studies show that the FAD gene family plays an important role in abiotic stress and drought often increases oil content [30, 31]. This finding suggests that drought and salt tolerance signaling pathways in *X. sorbifolium* may regulate lipid synthesis at the molecular level, this requires further systematic experimental validation.

## 5. Conclusion

In conclusion, 25 *XsFAD* genes were identified. By analyzing gene structure, *XsFAD* genes were divided into three subfamilies, FAB2, FAD2/FAD6, and ADS/SLD/DES, and three pairs of colinear genes were found. Through gene expression analysis, it was found that the expression of *XsFAD18* in FAD2 was high and significantly related to seed oil content. In future research, we will construct the genetic transformation system of *X. sorbifolium* and further verify the function of *XsFAD* genes. The molecular regulatory mechanisms in lipid synthesis will be revealed by joint analysis of the transcriptome and metabolomics. Then screen the key transcription factors by yeast hybridization and other experiments, and then analyze the promoter site of the transcription factor binding by ChIP and EMSA experiments. Meanwhile, we will design related stress experiments, such as drought stress and cold stress, to reveal the efficiency mechanism of environmental factors on oil production. This study provides a theoretical research basis and reference for the research of variety breeding, cultivation system, and genetic breeding of China Xinjiang *X. sorbifolium* Fruit.

## Supporting information

S1 Table Basic Information of 10 *X. sorbifolium* Germplasm.
(PDF)

S2 Table Coordinate Table of Matrix for Comprehensive Evaluation of 10 *X. sorbifolium* Germplasm.
(PDF)

S3 Table
Comprehensive Evaluation of $p_{i}^{2}$ of 10 *X. sorbifolium* Provenances.(PDF)
