# Supplementary material for: Genome-wide characterization of FAD gene family in Xanthoceras sorbifolium Bunge and germplasm assessment
Source: PLoS One. 2025 Mar 27;20(3):e0318900. doi: 10.1371/journal.pone.0318900 (PMC11949342; doi:10.1371/journal.pone.0318900)
Supplement: S1 Table — (PDF) [file pone.0318900.s001.docx]

**S1 Table. Basic Information of 10 *X. sorbifolium* Germplasm.**

| **ID** | **Fruit shape** | **fruit opening method** | **Average longitudinal diameter of fruit（mm）** | **Average transverse diameter of fruit (mm)** | **single fruit weight (g)** | **Seed size** | **seed diameter (mm)** | **Single fruit seeds number** | **thousand-seed weight（g）** | **Single fruit shell weight(g)** | **Shell thickness** | **Fruits number** | **Seed weight per plant（kg）** | **Seed oil content（%）** |
| --- | --- | --- | --- | --- | --- | --- | --- | --- | --- | --- | --- | --- | --- | --- |
|  |  |  |  |  |  |  |  |  |  |  | (mm) |  |  |  |
| **Liudong-1** | Peach shape | 3-lobed, less 4-lobed | 73.03 | 74.17 | 51.9 | big | 15.89 | 19-26 | 1197.1 | 25.69 | 7.67 | 103 | 2.589 | 63.49% |
| **Liudong-5** | Cylindrical | 3-lobed | 72.88 | 58.42 | 49.79 | big | 14.65 | 19 | 1230.8 | 23 | 6.73 | 195 | 5.036 | 56.10% |
| **80 acres-1** | sphericity | 3-lobed | 44.11 | 46.71 | 26.66 | small | 21.58 | 20 | 746 | 10.77 | 6.46 | 68 | 1.014 | 62.28% |
| **80 acres-5** | sphericity | 3-lobed | 47.85 | 50.1 | 27.51 | small | 21.1 | 16 | 800 | 13.01 | 6.11 | 62 | 0.793 | 69.45% |
| **80 acres-7** | sphericity | 3-lobed,less 4-lobed | 44.37 | 47.14 | 22.39 | small | 25.2 | 16 | 788 | 15.7 | 4.67 | 58 | 0.731 | 65.88% |
| **49-4** | sphericity | 3-lobed and 4-lobed | 68.43 | 70.61 | 48.22 | big | 14.37 | 19 | 994 | 16.12 | 5.32 | 88 | 1.662 | 57.11% |
| **80-3** | stylolitic | 3-lobed | 58.34 | 63.19 | 47.43 | medium | 15.73 | 18 | 830 | 17.19 | 6.21 | 79 | 1.18 | 64.12% |
| **80-3-2** | Peach shape | 3-lobed | 49.43 | 52.67 | 41.28 | small | 13.79 | 18 | 792 | 18.23 | 5.31 | 102 | 1.454 | 69.05% |
| **81-6-1** | Peach shape | 3-lobed | 48.26 | 54.56 | 40.37 | small | 13.76 | 19 | 716 | 17.61 | 5.27 | 121 | 1.348 | 66.09% |
| **131-75** | Peach shape | 3-lobed | 45.23 | 52.89 | 42.63 | small | 12.78 | 18 | 766 | 19.36 | 4.56 | 303 | 4.177 | 72.45% |
