# Supplementary material for: Genome-wide characterization of FAD gene family in Xanthoceras sorbifolium Bunge and germplasm assessment
Source: PLoS One. 2025 Mar 27;20(3):e0318900. doi: 10.1371/journal.pone.0318900 (PMC11949342; doi:10.1371/journal.pone.0318900)
Supplement: S2 Table — (PDF) [file pone.0318900.s002.docx]

**S2 Table. Coordinate Table of Matrix for Comprehensive Evaluation of 10 *X. sorbifolium* Germplasm.**

| **ID** | **Average longitudinal diameter of fruit（mm）** | **Average transverse diameter of fruit (mm)** | **single fruit weight (g)** | **seed diameter (mm)** | **Single fruit seeds number** | **thousand-seed weight(g)** | **Single fruit shell weight(g)** | **Shell thickness** | **Fruits number** | **Seed weight per plant（kg）** | **Seed oil content（%）** |
| --- | --- | --- | --- | --- | --- | --- | --- | --- | --- | --- | --- |
|  |  |  |  |  |  |  |  | **(mm)** |  |  |  |
| **Liudong-1** | 1.000 | 1.000 | 1.000 | 0.736 | 1.000 | 0.973 | 1.000 | 1.000 | 0.340 | 0.514 | 0.876 |
| **Liudong-5** | 0.998 | 0.788 | 0.959 | 0.679 | 0.864 | 1.000 | 0.895 | 0.877 | 0.644 | 1.000 | 0.774 |
| **80 acres-1** | 0.604 | 0.630 | 0.514 | 1.000 | 0.909 | 0.606 | 0.419 | 0.842 | 0.224 | 0.201 | 0.860 |
| **80 acres-5** | 0.655 | 0.675 | 0.530 | 0.978 | 0.727 | 0.650 | 0.506 | 0.797 | 0.205 | 0.157 | 0.959 |
| **80 acres-7** | 0.608 | 0.636 | 0.431 | 1.168 | 0.727 | 0.640 | 0.611 | 0.609 | 0.191 | 0.145 | 0.909 |
| **49-4** | 0.937 | 0.952 | 0.929 | 0.666 | 0.864 | 0.808 | 0.627 | 0.694 | 0.290 | 0.330 | 0.788 |
| **80-3** | 0.799 | 0.852 | 0.914 | 0.729 | 0.818 | 0.674 | 0.669 | 0.810 | 0.261 | 0.234 | 0.885 |
| **80-3-2** | 0.677 | 0.710 | 0.795 | 0.639 | 0.818 | 0.643 | 0.710 | 0.692 | 0.337 | 0.289 | 0.953 |
| **81-6-1** | 0.661 | 0.736 | 0.778 | 0.638 | 0.864 | 0.582 | 0.685 | 0.687 | 0.399 | 0.268 | 0.912 |
| **131-75** | 0.619 | 0.713 | 0.821 | 0.592 | 0.818 | 0.622 | 0.754 | 0.595 | 1.000 | 0.829 | 1.000 |
