# Supplementary material for: Genome-wide characterization of FAD gene family in Xanthoceras sorbifolium Bunge and germplasm assessment
Source: PLoS One. 2025 Mar 27;20(3):e0318900. doi: 10.1371/journal.pone.0318900 (PMC11949342; doi:10.1371/journal.pone.0318900)
Supplement: S3 Table — (PDF) [file pone.0318900.s003.docx]

**S3 Table. Comprehensive Evaluation of**
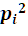
 **of 10 *X. sorbifolium* Provenances.**

| **ID** | **Average longitudinal diameter of fruit（mm）** | **Average transverse diameter of fruit (mm)** | **single fruit weight (g)** | **seed diameter (mm)** | **Single fruit seeds number** | **thousand-seed weight（g）** | **Single fruit shell weight(g)** | **Shell thickness** | **Fruits number** | **Seed weight per plant（kg）** | **Seed oil content（%）** | $\sum{\boldsymbol{p}_{\boldsymbol{i}}}^{\boldsymbol{2}}$ | **rank** |
| --- | --- | --- | --- | --- | --- | --- | --- | --- | --- | --- | --- | --- | --- |
|  |  |  |  |  |  |  |  | (mm) |  |  |  |  |  |
| **Liudong-1** | 0.000 | 0.000 | 0.000 | 0.070 | 0.000 | 0.001 | 0.000 | 0.000 | 0.436 | 0.236 | 0.015 | 0.757 | 2 |
| **Liudong-5** | 0.000 | 0.045 | 0.002 | 0.103 | 0.019 | 0.000 | 0.011 | 0.015 | 0.127 | 0.000 | 0.051 | 0.372 | 1 |
| **80 acres-1** | 0.157 | 0.137 | 0.237 | 0.000 | 0.008 | 0.155 | 0.337 | 0.025 | 0.602 | 0.638 | 0.020 | 2.315 | 9 |
| **80 acres-5** | 0.119 | 0.105 | 0.221 | 0.000 | 0.074 | 0.123 | 0.244 | 0.041 | 0.633 | 0.710 | 0.002 | 2.272 | 8 |
| **80 acres-7** | 0.154 | 0.133 | 0.323 | 0.028 | 0.074 | 0.129 | 0.151 | 0.153 | 0.654 | 0.731 | 0.008 | 2.539 | 10 |
| **49-4** | 0.004 | 0.002 | 0.005 | 0.112 | 0.019 | 0.037 | 0.139 | 0.094 | 0.503 | 0.449 | 0.045 | 1.408 | 4 |
| **80-3** | 0.040 | 0.022 | 0.007 | 0.073 | 0.033 | 0.106 | 0.109 | 0.036 | 0.547 | 0.586 | 0.013 | 1.574 | 5 |
| **80-3-2** | 0.104 | 0.084 | 0.042 | 0.130 | 0.033 | 0.127 | 0.084 | 0.095 | 0.440 | 0.506 | 0.002 | 1.648 | 6 |
| **81-6-1** | 0.115 | 0.070 | 0.049 | 0.131 | 0.019 | 0.175 | 0.099 | 0.098 | 0.361 | 0.536 | 0.008 | 1.661 | 7 |
| **131-75** | 0.145 | 0.082 | 0.032 | 0.166 | 0.033 | 0.143 | 0.061 | 0.164 | 0.000 | 0.029 | 0.000 | 0.855 | 3 |
